# The Role of Branched Chain Ketoacid Dehydrogenase Kinase (BCKDK) in Skeletal Muscle Biology and Pathogenesis

**DOI:** 10.3390/ijms25147601

**Published:** 2024-07-11

**Authors:** Joshua Fernicola, Sagar Vyavahare, Sonu Kumar Gupta, Aditya Kalwaghe, Kate Kosmac, Adam Davis, Matthew Nicholson, Carlos M. Isales, Rahul Shinde, Sadanand Fulzele

**Affiliations:** 1 Department of Medicine, Division of Endocrinology, Augusta University, Augusta, GA 30912, USA; jfernicola@augusta.edu (J.F.); svyavahare@augusta.edu (S.V.); sgupta2@augusta.edu (S.K.G.); kalwagheaditya45@gmail.com (A.K.); adavis5@augusta.edu (A.D.); matnicholson@augusta.edu (M.N.); cisales@augusta.edu (C.M.I.); 2 Department of Physical Therapy, Augusta University, Augusta, GA 30912, USA; kkosmac@augusta.edu; 3 Center for Healthy Aging, Augusta University, Augusta, GA 30912, USA; 4 Immunology, Microenvironment and Metastasis Program, The Wistar Institute Cancer Center, Philadelphia, PA 19104, USA; rshinde@wistar.org; 5 Department of Cell Biology and Anatomy, Augusta University, Augusta, GA 30912, USA

**Keywords:** BCKDK, skeletal muscle, muscle pathogenesis

## Abstract

Muscle wasting can be caused by nutrition deficiency and inefficient metabolism of amino acids, including Branched Chain Amino Acids (BCAAs). Branched Chain Amino Acids are a major contributor to the metabolic needs of healthy muscle and account for over a tenth of lean muscle mass. Branched chain alpha-ketoacid dehydrogenase (BCKD) is the rate limiting enzyme of BCAA metabolism. Inhibition of BCKD is achieved through a reversible phosphorylation event by Branched Chain a-ketoacid dehydrogenase kinase (BCKDK). Our study set out to determine the importance of BCKDK in the maintenance of skeletal muscle. We used the Gene Expression Omnibus Database to understand the role of BCKDK in skeletal muscle pathogenesis, including aging, muscular disease, and interrupted muscle metabolism. We found BCKDK expression levels were consistently decreased in pathologic conditions. These results were most consistent when exploring muscular disease followed by aging. Based on our findings, we hypothesize that decreased BCKDK expression alters BCAA catabolism and impacts loss of normal muscle integrity and function. Further research could offer valuable insights into potential therapeutic strategies for addressing muscle-related disorders.

## 1. Introduction

Age-related decline in bone and muscle health is a widely accepted yet incompletely understood phenomenon. Several studies have shown that after the fourth decade of life, there is a near continuous decrease in lean muscle mass and bone density [1]. The rate of muscle synthesis is also lower in older populations, which further exacerbates the decrease in mechanical functionality that coincides with age [2]. This degeneration has an overwhelming effect on the well-being of patients and can lead to instability, injury, disability, and physical dependence [3,4]. Recent studies demonstrated that alterations in nutritional signaling pathways and amino acid metabolism are major contributor in the pathogenesis of muscle loss. Amino Acids, including Branched Chain Amino Acids (BCAAs), play a vital role in muscle development, as well as maintenance of the integrity of muscle fibers [5].

BCAAs comprise three of the nine essential amino acids. These three amino acids, valine, leucine, and isoleucine, make up approximately 14% of lean muscle mass [5]. In addition, BCAAs and intermediate metabolites also play an important role in protein synthesis [6,7,8]. BCAA metabolism is tightly controlled by a series of pathways and enzymes specific to their branched R group. The rate-limiting step of the main metabolic pathway of BCAAs occurs in the mitochondrial matrix of cells. This step regulates the activity of the entire pathway and is catalyzed by the enzyme complex Branched chain a-ketoacid dehydrogenase (BCKDc). Differential metabolism of BCAAs is accomplished through the activity of a regulatory kinase of BCKDc (BCKDK) [5,9], which is expressed in a tissue specific manner [10]. BCKDK inactivates BCKDc through a reversible phosphorylation event, which, in turn, inhibits BCAA utilization. Since BCAAs exert many different cellular effects, the activity of BCKDK remains vital for homeostasis, especially in tissues like skeletal muscle that rely heavily on BCAAs for signaling and structure [7]. In these tissues, the maintenance of BCAAs at an appropriate level is crucial for cellular development. Prior work in osteoclasts by Go et al. has shown that intermediate levels of BCAAs (400 μM) promote osteoclast differentiation while higher concentrations (800 μM) tend to inhibit it [11]. This finding, along with the high content of BCAAs in muscle tissue, makes the metabolism of BCAAs a potential pathway of interest regarding muscle health.

While the function of BCKDK is well known, limited knowledge is available on the correlation between the expression of BCKDK and muscle-related pathogenesis. Based on the literature, we hypothesized that BCKDK expression plays an important role in muscle biology, and aberrant expression might lead to muscle pathology. In this study, we summarize the role of BCKDK in muscle biology and correlate its expression with pathological phenotype using the Gene Expression Omnibus (GEO) data set. We identified several muscle-related pathologies in which BCKDK expression was dysregulated.

## 2. Results

### 2.1. Alteration in the BCKDK Gene Expression between Young and Old Human Skeletal Muscle

The data were sourced from the GEO dataset (GEO ID 1592758) for the males and GEO dataset (GEO ID 3242858) for females. The skeletal muscle samples were taken from the vastus lateralis, and gene expression analysis was conducted using high-density oligonucleotide arrays (Affymetrix HG-U133A and HF-U133B). We compare the expression of the BCKDK gene between young and old individuals. The data demonstrated a significant (*p*-value = 0.0148) decrease in the BCKDK gene expression in females. We found no changes in the male GEO data set.

### 2.2. BCKDK Gene Expression in Pathological Conditions in Skeletal Muscle

Gene expression analysis of the BCKDK gene was conducted using GEO datasets (GEO ID 127864049, GEO ID 26550958, and GEO ID 56390258) in the pathological condition. The BCKDK gene expression was examined in human skeletal muscle biopsies from rhabdomyosarcoma patients compared to healthy controls, revealing a significant (*p*-value < 0.0001) decrease in gene expression. Similarly, the GEO ID 56390258 data set was used to analyze BCKDK gene expression in skeletal muscle biopsies from patients with juvenile dermatomyositis. The data demonstrated a significant (*p*-value < 0.0001) decrease in the BCKDK gene expression compared to control. The GEO (ID 26550958) dataset BCKDK gene expression in Dermatomyositis patients and healthy controls did not show a change in expression.

### 2.3. BCKDK Expression in Compromised Sarcomere Structure

Gene expression data obtained from mice models were collected from three GEO datasets: GEO ID 132032924, GEO ID 1110645, and GEO ID 46043025 from the different knockout animals. The dataset with ID 132032924 was sourced from Li et al.’s GEO dataset, showing a significant (*p*-value = 0.01) decrease in BCKDK gene expression in the nebulin KO compared to wild-type [12]. The dystrophin-deficient (skeletal muscle) mice data (GEO ID 1110645) were retrieved from Tseng et al.’s dataset, showing a significant decrease (*p*-value = 0.04) in the BCKDK gene expression [13]. We also analyzed the cardiac muscles of the dystrophin-deficient mice (GEO ID 46043025). The data showed a significant (*p*-value = 0.01) decrease in the BCKDK gene expression compared to corresponding controls.

### 2.4. Alteration of BCKDK Gene Expression Following Interruption in Normal Metabolic Activity

The alteration in BCKDK gene expression was investigated in a Mus Musculus model under various conditions using data from different GEO datasets. We did not find any changes in the (GEO ID 63213636) myostatin depletion, (GEO ID 59767219) myostatin inhibition, and (GEO ID 22494325) AMP protein kinase knockout analysis of BCKDK gene expression in skeletal muscle of Mus Musculus. We found significant upregulation of BCKDK gene expression in skeletal muscles of pyruvate supplementation compared to controls from the data from GEO ID 29303836.

## 3. Discussion

Skeletal Muscle constitutes approximately one-third of total body mass [14] and serves multiple functions, including thermoregulation, physical protection, and voluntary movement. Skeletal muscle is a striated tissue comprised of repeating contractile units known as sarcomeres. The proteins myosin, tropomyosin, actin, and titin are arranged in a manner that enables overlap of the elements and global shortening of muscle fibers [15]. Due to the high protein content of skeletal muscle, amino acids are essential for meeting tissue’s anabolic and catabolic demands. BCAAs comprise over 10% of muscle mass and provide approximately 35% of healthy muscle’s essential amino acid requirements [16]. Therefore, these amino acids and enzymes involved in their metabolism are crucial in muscle physiology and pathobiology.

### 3.1. Aging and BCKDK Gene Expression

Human connective tissue health is a dynamic system influenced by a complex interplay of environmental factors and genetic predisposition. Despite several proven modifiable behaviors that can enhance skeletal muscle strength, research on aging consistently indicates a gradual decrease in muscle mass with age [17,18]. One of the hypotheses to explain this decline is that the metabolic processes responsible for maintaining connective tissue health become less effective as we age. In two separate studies by Welles et al., biopsies from the vastus lateralis of age-matched men and women were collected to determine changes in gene expression [2,19].

**Figure 1:**
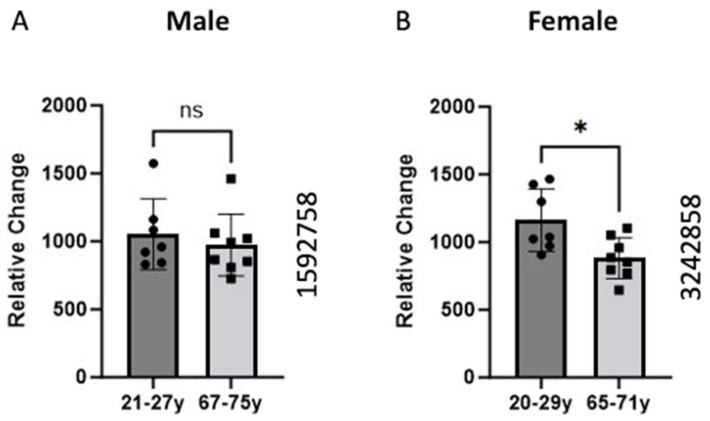
Comparison of BCKDK gene expression between young and old age groups in Male (**A**) and Female (**B**) groups. Skeletal muscle biopsies of the vastus lateralis were obtained from human participants and gene expression was analyzed using Affymetrix (Santa Clara, CA, USA) HG-U133A and HF-U133B high-density oligonucleotide arrays. The data for (**A**) were retrieved from GEO dataset uploaded by Welle et al. GEO ID 1592758) (*n* = 7–8) [2]. Identical experimental procedures were used to assess female gene expression. This data from (**B**) were retrieved from GEO dataset uploaded by Welle et al. (GEO ID 3242858) significance determined by GEO2R adjusted * *p*-value = 0.0148 (*n* = 7–8) [19].

Data on BCKDK expression shows decreased expression in males and females with age. GEO data analysis demonstrated a significant (*p* = 0.0148) decrease in BCKDK gene expression in the females and a non-significant decline in males (Figure 1). The divergence between men and women could be attributed to body composition and metabolism variations between the sexes. Specifically, men typically possess a higher proportion of lean muscle than females [20]. This suggests that alterations to skeletal muscle metabolism may be more prominent in maintaining muscle health in females than in males. This suggested that the phenomenon occurs in both genders but is more substantial in females, possibly due to their lower baseline levels of lean mass.

Previous work also shows that men and women utilize carbohydrates, proteins, and lipids at different rates at rest and during exercise [21]. One group showed that men exhibit higher rates of carbohydrate and leucine oxidation while women had higher rates of lipid oxidation [22]. Estrogen is one hormone that can partly explain this phenomenon. One study showed supplementing men with estrogen reduced whole-body leucine oxidation [22]. Another group studying rats found that female rats express higher amounts of BCKDK when compared to their male counterparts [23]. Both findings suggest Estrogen plays an important role in the regulation of BCKDK. This would explain greater decreases in BCKDK gene expression in females (Figure 1). Due to post-menopausal changes, the aged (65–71-year-old) women likely have lower estrogen than the young (20–29-year-old group).

### 3.2. BCKDK Gene Expression in Pathological Conditions in Skeletal Muscle

**Figure 2:**
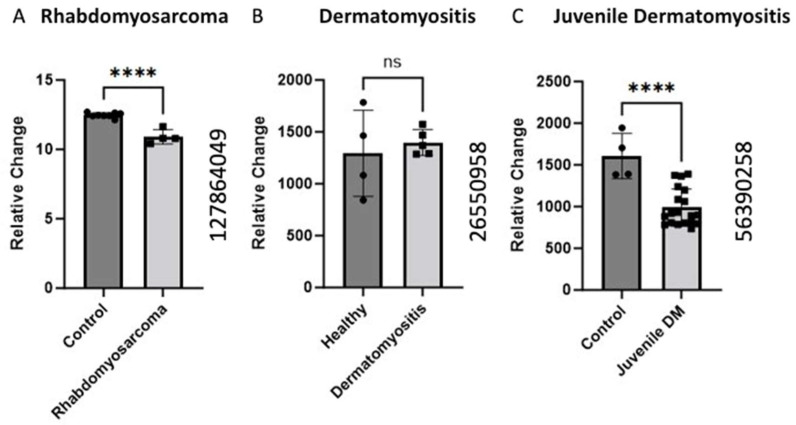
Alteration of BCKDK gene expression in pathological condition in skeletal muscle (**A**). The data for (**A**) were retrieved from GEO dataset uploaded by Sher et al. GEO ID 127864049) significance determined by GEO2R adjusted **** *p*-value < 0.0001 [24]. (*n* = 8–4). (**B**) BCKDK gene expression assessed using human skeletal muscle biopsises from patients with Dermatomyositis compared with healthy controls. The data for (**B**) were retrieved from GEO dataset (GEO ID 26550958) (*n* = 4–5). (**C**) BCKDK gene expression assessed using skeletal muscle biopsies from patients with juvenile dermatomyositis compared with healthy controls. The data were retrieved from GEO dataset uploaded by Chen et al. (GEO ID 56390258) significance determined by GEO2R adjusted **** *p*-value < 0.0001 (*n* = 4–19) [27].

We also collected data on various muscle pathological conditions that could negatively impact muscle health apart from age. Sher et al. performed gene expression studies on rhabdomyosarcoma muscle samples [24]. Rhabdomyosarcoma is a high-grade neoplasm of skeletal muscle precursor cells that is the most common pediatric soft tissue sarcoma [25]. The data showed significantly decreased levels of BCKDK expression in the experimental mice compared to healthy controls (Figure 2). BCAAs are essential nutrients for cancer cells, with some studies even using BCAA metabolic enzymes as diagnostic markers of cancer [26]. Decreased inhibition by BCKDK would allow dysregulated cells, such as sarcomas, to proliferate quicker than the native cells.

Additionally, we identified two studies on gene expression in dermatomyositis. Dermatomyositis is an autoimmune idiopathic inflammatory myopathy characterized by inflammation, cutaneous manifestations, and increased risk of certain cancers [28]. Data collected on juvenile dermatomyositis (JDM) [27] and adult dermatomyositis showed a decrease in BCKDK expression. The JDM data showed significant (*p* < 0.0001) changes in BCKDK expression, but no significant difference was observed for the DM model, potentially due to the low sample size (*n* = 4–5). JDM patients experience increased inflammation and decreased muscle mass and strength [29]. Similar results regarding decreased muscle mass were discovered in skeletal muscle isolated from mice with BCKDK gene deletion [30]. Although the mechanisms of DM and JDM are poorly understood, this gene expression data may partly explain the muscular pathology common to these conditions.

### 3.3. BCKDK Expression in Compromised Sarcomere Structure or Function

**Figure 3:**
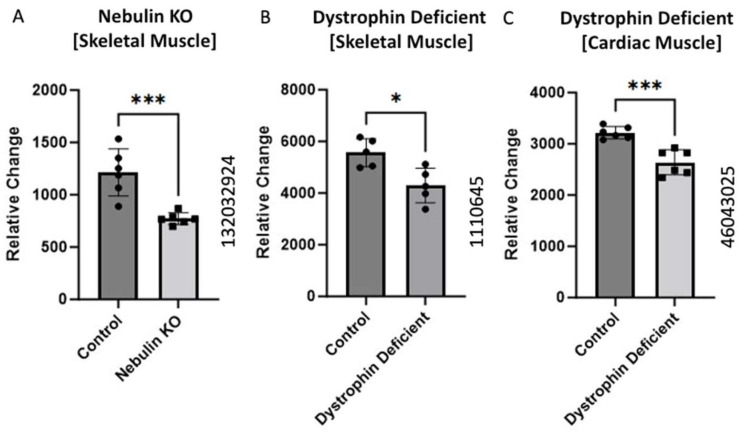
BCKDK expression in compromised Sarcomere structure or function. Data from (**A**) (GEO ID 132032924), (**B**) (GEO ID 1110645), and (**C**) (GEO ID 46043025) were collected using a Mus Musculus model. The data for (**A**) were retrieved from GEO dataset uploaded by Li et al. and significance determined by GEO2R adjusted *** *p*-value = 0.01 (*n* = 6) [12]. The data for (**B**) were retrieved from GEO dataset uploaded by [13] and significance determined by GEO2R adjusted * *p*-value < 0.04. *n* = 5. The data for (**C**) were retrieved from the GEO database and significance was determined by GEO2R adjusted *** *p*-value = 0.01 (*n* = 6).

The sarcomere is the contractile apparatus of muscle [31]. It is the functional unit of muscle tissue responsible for muscle contraction and is comprised of various proteins, including actin and myosin, arranged in a highly organized structure. During muscle contraction, the sarcomere shortens as the actin and myosin filaments slide past each other, generating force and causing muscle contraction [31]. This process allows muscles to generate tension and perform various movements in the body. The sarcomere is the structural unit of muscle that undergoes significant changes and damage in Duchenne Muscular Dystrophy [32]. We analyzed the data from three different transgenic mice with knockouts for different muscle-related proteins (Figure 3). Two studies represent a pseudo-Duchenne Muscular Dystrophy DMD model, as the lack of the dystrophin protein accounts for the pathology of DMD [33].

All three studies showed significantly decreased BCKDK expression in the pathological condition compared to the respective controls. Nishikawa et al. uncovered a unique interplay between nebulin and BCAA supplementation [34]. They found BCAA supplementation plays a strong inhibitory role in muscle atrophy. They proposed that increased BCAA aids in the regeneration of nebulin and increase the phosphorylation of an accessory protein known as Neural-Wiskott Aldrich Syndrome Protein (N-WASP) [34]. Both nebulin and N-WASP are important elements of the thin filaments of sarcomeres. It is possible that in the nebulin knockout model, BCKDK is downregulated to increase the catabolic flux of BCAAs. This elevation of BCAA utilization may increase N-WASP phosphorylation to compensate for the absence of nebulin content in thin filaments. These data present another mechanism by which BCKDK can modify muscle health.

The data from the two dystrophin knockout studies present a similar trend. Past work in DMD shows that muscle fibers are more prone to induced injury when lacking functional dystrophin [33]. Alternatively, BCAA utilization improves both hypertrophy and maintenance of muscle fibers [35]. Decreased expression of BCKDK in compromised muscle fibers could protect against myofibril degradation through increased BCAA catabolism. This could help explain the decrease gene expression of BCKDK in both dystrophin knockout mice.

### 3.4. Myostatin and BCKDK

**Figure 4:**
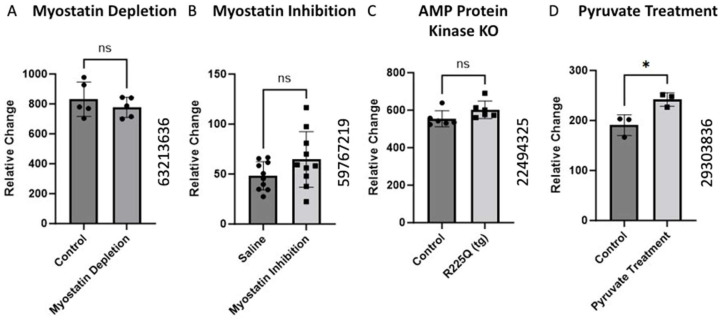
Alteration of BCKDK gene expression following interruption in normal metabolic activity. The data for (**A**) were retrieved from GEO dataset using a Mus Musculus model uploaded by Welle et al. (GEO ID 63213636) (*n* = 5) [37]. (**B**) BCKDK gene expression assessed using skeletal muscle biopsises from Mus Musculus with inhibited myostatin activity. The data for (**B**) were retrieved from GEO dataset (GEO ID 59767219) (*n* = 10). (**C**) BCKDK gene expression assessed using skeletal muscle biopsies from transgenic Mus Musculus model with AMP Protein Kinase gene knockout compared with healthy controls. The data for (**C**) were retrieved from GEO dataset uploaded by Nilsson et al. (GEO ID 22494325) (*n* = 6) [38]. The data for (**D**) corresponds to research in a Mus Musculus model in which muscle cells from a pyruvate supplementation group were compared to controls. The data were retrieved from work uploaded by Wilson et al. (GEO ID 29303836) significance determined by GEO2R adjusted * *p*-value < 0.05 (*n* = 3) [39].

Myostatin is a Transforming Growth Factor-Beta (TGF-β) protein that negatively regulates skeletal muscle growth [36]. We identified two studies inhibiting myostatin in a mouse model (Figure 4A,B). The expression of BCKDK moved in opposite directions in each study, but neither change was statistically significant. Based on these data, myostatin activity appears to have a minimal effect on BCKDK expression.

AMP-activated Protein Kinase (AMPK) is a vital metabolic sensor triggered by low-energy states. When activated, AMPK stimulates catabolic processes that produce ATP [40]. Gene expression data from an experiment involving transgenic AMPK knockout mice showed no significant change in the BCKDK expression (Figure 4C). AMPK typically antagonizes the action of BCKDK through protein signaling cascades. Thus, the absence of AMPK would be expected to increase BCKDK expression. Although the change in the gene expression was not significant, there was a slight increase in BCKDK expression. Further studies are required to rule out interactions between AMPK and BCKDK.

Chaudhry et al. examined the effects of pyruvate supplementation in mice. Pyruvate is a simple acid that is the end product of glycolysis and an active part of many metabolic pathways [41]. The data showed pyruvate supplementation increased the BCKDK gene’s expression (Figure 4D). This increase may be mediated by an AMPK-related mechanism. Increased concentrations of glycolytic products, like pyruvate, simulate a fed state which will inhibit catabolic processes [42]. The increase in BCKDK expression would be a way for cells to slow BCAA catabolism. While BCAAs and muscle health are large areas of interest, the literature lacks extensive studies on the role of BCKDK. As such, direct relationships between BCKDK and muscle health will require further attention from the scientific community. In addition, gene expression is a dynamic process that can be influenced by factors such as age, nutrition, aerobic conditioning, body composition, etc. Prior work has demonstrated the importance of BCAAs in skeletal muscle; however, the intricacies of muscle homeostasis are yet to be completely understood. Given the significant role of BCKDK in BCAA metabolism, a comprehensive understanding of muscle physiology necessitates further investigation into the processes mediated by BCKDK.

Our study has some limitations, such as a small sample size, the utilization of GEO data sets from animal models, lack of longitudinal data, and detailed nutritional information. Establishing only a direct cause-and-effect relationship between BCKDK and muscle health requires further mechanistic studies. Our study lacks longitudinal data, which could provide insights into the dynamic changes in BCKDK expression and muscle health over time. The study lacks detailed information on diets or nutritional status, which could potentially influence BCKDK expression and muscle physiology.

## 4. Methods

Based on our hypothesis, we used the Gene Expression Omnibus (GEO) database to search for available data on our enzyme of interest. The enzyme BCKDK plays an important modulatory role in the metabolism of BCAAs. To evaluate muscle and bone effects potentially related to BCKDK, we used search results such as “sarcopenia”, “aging”, “muscle”, and “connective tissue”. Studies in animal models and alternate cell lines were also considered so as not to miss any significant data points. Once appropriate studies were identified, we compiled data to assess notable differences in gene expression between control and experimental groups. All utilized data were evaluated for statistical significance using t-testing and analyzed by other metrics, including date published and test design. The GEO database allowed our group to synthesize data that may have gone unused to challenge and or bolster our hypothesis. Details of relevant studies’ designs can be found in the Table 1.

## 5. Conclusions

Our study reveals a consistent association between decreased BCKDK expression and pathologic conditions impacting skeletal muscle, with particular relevance to muscular disease and aging models. BCKDK acts as an inhibitor of BCKD leading to decreased oxidation of BCAAs. This implies that reduced BCKDK levels could enhance BCAA breakdown and could contribute significantly to muscle wasting and the decline of muscle integrity and function. While this mechanism is not completely understood, it is possible that excessive breakdown of BCAAs could generate metabolites that negatively impact skeletal muscle. Alternatively, since BCAAs are vital for protein synthesis, their depletion through enhanced oxidation could impair structural integrity and muscle growth. In pathological conditions, decreased BCKDK expression may serve as a compensatory mechanism to generate energy for compromised tissues by enhancing BCAA oxidation. The trends in BCKDK gene expression across multiple studies suggest a link between this enzyme and normal muscle function. Pathologic conditions exhibited significantly decreased BCKDK gene expression in most of the studies presented in this paper. Muscle homeostasis is a complicated process that extends much further than the influence of a single enzyme. However, elucidating the role BCKDK plays in this process could assist in generating therapies for muscular dysfunction.

## Figures and Tables

**Table 1:** Details of the GEO dataset study designs used to examine BCKDK gene expression in different models.

S. No	GEO Database Identification No.	Treatment	Study Design	Model
1	3242858	Age compared	Needle biopsies of the vastus lateralis of the left leg were collected from seven 20–29 yo females and eight 65–71 yo females. RNA was extracted from the muscle and reverse transcribed to cDNA with oligo-dT-[T7] as the primer. The cDNA was then converted to cRNA and probed using Affymetrix (Santa Clara, CA, USA) HG-U133A and HF-U133B high-density oligonucleotide arrays. These probes measure expression of ~30,000 genes. Expression was then compared between groups [19].	Human
2	56390258	Juvenile Dermatomyositis (JDM)	Skeletal muscle biopsies were gathered from two groups of adolescent females with untreated juvenile dermatomyositis (JDM). The first group consisted of three girls with untreated JDM of <2 months duration. The second group consisted of sixteen girls with untreated JDM of >2 months duration. Total RNA was isolated from the samples and gene expression was assessed using the Affymetrix HG-U133A probe. A Welch t-test was then used to calculate the probabilities of significant gene expression changes between samples [27].	Human
3	26550958	Adult Dermatomyositis (DM)	This data set contains gene data from muscle biopsies gathered from four women with untreated dermatomyositis. A control group of five healthy volunteers were used to assess intergroup differences. Biopsies were analyzed using Affymetrix Human Genome U133A Array. Further information regarding this data set can be found under GEO series accession number GSE5370.	Human
4	127707736	5-azacytidine on mesenchymal progenitors	In this study by Zhou et al., Mesenchymal stem cell lines were obtained from a group of 6–8-week-old ICR mice. The cells were seeded on T-175 flasks and allowed to populate for 24 h. MSC’s were collected when they had grown to 90% confluence. Isolated cells were cultured in 96-well plates at a concentration of 2 × 10^3^ cells/well and treated with differing concentrations of 5-azacytidine, a DNA methylation inhibitor. Treated cells were then removed and assessed for osteogenic gene-expression and calcium mineralization activity [43].	Mus-Musculus
5	60459336	Pyruvate supplementation in myoblast	In this study by Wilson et al., myoblasts from mice were obtained and grown in media with and without pyruvate. This work looked into the relationship between pyruvate supplementation and mitochondrial functioning in vitro. After cells had been treated, they were collected for flow cytometry, confocal microscopy, protein expression studies, gene chip analysis, and more. The results of the gene chip analysis were deposited in the National Center for Biotechnology Information (NCBI) Gene Expression Omnibus (GEO) and are accessible through GEO series accession number GSE5497.	Mus-Musculus
6	46043025	Dystrophin Deficient Cardiomyocyte	In this study by JD porter, a line of dystrophin knockout mice was used to study the functioning of the heart in a Duchene Muscular Dystrophy model. Samples of cardiac myocytes were gathered at 8 weeks and 10.5 months from wild type mice and the dystrophin knockout mice. These samples were then used to analyze gene expression to elucidate intergroup differences. The results of the gene chip analysis were deposited in the National Center for Biotechnology Information (NCBI) Gene Expression Omnibus (GEO) and are accessible through GEO series accession number GSE1471.	Mus-Musculus

## Data Availability

The data that support the findings of this study are available from the corresponding author upon reasonable request.
